# Optimization of Multiparameters for Increased Yields of Cytochrome B5 in Bioreactors

**DOI:** 10.3390/molecules26144148

**Published:** 2021-07-08

**Authors:** Ricardo F. S. Pereira, Carla C. C. R. de Carvalho

**Affiliations:** 1 Department of Bioengineering, iBB—Institute for Bioengineering and Biosciences, Instituto Superior Técnico, Universidade de Lisboa, Av. Rovisco Pais, 1049-001 Lisboa, Portugal; ricardofspereira@tecnico.ulisboa.pt; 2 Associate Laboratory i4HB—Institute for Health and Bioeconomy, Instituto Superior Técnico, Universidade de Lisboa, Av. Rovisco Pais, 1049-001 Lisboa, Portugal

**Keywords:** fermentation, recombinant protein, cytochrome b5, bioreactor, bioprocess, scale up, partial least squares, PID controllers

## Abstract

The production of recombinant proteins is gaining increasing importance as the market requests high quality proteins for several applications. However, several process parameters affect both the growth of cells and product yields. This study uses high throughput systems and statistical methods to assess the influence of fermentation conditions in lab-scale bioreactors. Using this methodology, it was possible to find the best conditions to produce cytochrome b5 with recombinant cells of *Escherichia coli*. Using partial least squares, the height-to-diameter ratio of the bioreactor, aeration rate, and PID controller parameters were found to contribute significantly to the final biomass and cytochrome concentrations. Hence, we could use this information to fine-tune the process parameters, which increased cytochrome production and yield several-fold. Using aeration of 1 vvm, a bioreactor with a height-to-ratio of 2.4 and tuned PID parameters, a production of 72.72 mg/L of cytochrome b5 in the culture media, and a maximum of product to biomass yield of 24.97 mg/g could be achieved.

## 1. Introduction

Cytochrome b5 (cyt b5) is a hemoprotein (also hemeprotein or hemoprotein) first described in *Platysumia cecropia* in 1950 [1]. Since then, it has been described in bacteria, virus, fungi, plants, and animals, and it has been suggested that it appeared very early in evolution [2,3,4,5]. The small electron transport protein has important roles for the successful desaturation of fatty acids, reduction of cytochrome P450 and modulation of its activity, and for changing of ferric iron to ferrous iron in hemoglobin in erythrocytes [2,3,6,7,8]. Hepatic cyt b5 contains a soluble hydrophilic core where the heme group is present, and a membrane anchoring tail of hydrophobic nature [6].

In mammals, two cyt b5 homologous isoforms can be found, both at membrane level: One is anchored in the membrane of the endoplasmic reticulum, and the other is in the outer membrane of mitochondria [9,10]. Microsomal cyt b5 is involved in electron transport to several systems, such as cytochrome P450, different oxygenases, and desaturases, and cyt b5 reductases [2,11]. The latter is a family of flavoproteins that participate in multiple metabolic conversions, including elongation and desaturation of fatty acids, improvement of mitochondrial function, protection from oxidative damage, and inhibition of chronic pro-inflammatory pathways [12,13]. There has been evidence that cyt b5 and cyt b5 reductase can be the sole electron donors of the hepatic cytochrome P450 system [14], which could have implications, e.g., in the metabolism of carcinogens [15], xenobiotic hydroxylamines, and amidoximes [16], and pharmaceuticals including anticancer drugs [17].

The production of cyt b5 in bioreactors may provide sufficient amounts for its application in several research and development studies, namely: In vitro studies of lipid-protein interactions [18]; determination of the effect of allosteric alteration of cyt b5 active site conformation [19]; studies regarding cyt b5 interactions with cytochrome P450 enzymes aimed at activation of pharmaceutical drugs [20,21]; drug metabolism and biosynthesis of natural products by the P450-cyt b5 complex and development of corresponding pharmacokinetic models to predict in vivo action in the early stages of drug discovery [22,23]. Cyt b5 has also been successfully used as an efficient affinity matrix for the purification of electron-transfer proteins [24], and as a model protein for developing batch and continuous extraction processes [25].

Only a few studies regarding the cyt b5 production in bioreactor have been published [26,27]. It was reported that increased stirring rates and the increased air pressure inside the reactor could increase cyt b5 productivity [26]. Currently, the production of recombinant proteins is a multibillion-dollar market [28]. *Escherichia coli* is considered the most efficient expression host for a large number of therapeutical recombinant proteins, due to its well-studied genetics, cell biology, metabolism, easy handling, safety, and the possibility of achieving high production yields at low cost [29,30].

Recent developments in bioprocessing of recombinant proteins include, e.g., the use of high-throughput devices for optimizing conditions, Design of Experiments techniques, and integrated continuous bioprocessing [31,32,33,34]. Strategic improvements in the producing strain and in the expression system, the culture medium, and cultivation conditions may be optimized [35,36,37]. Among the parameters tested in this work are the carbon and nitrogen sources type and concentration, aeration and stirring rates, pH, and the less commonly studied height-to-diameter ratio of the bioreactors and the algorithms controlling the oxygen sensors. The optimization of the conditions allowing high production yields of hepatic cyt b5 by *E. coli* TB-1 cells were carried out by multivariate statistical techniques using data collected in high throughput systems and in lab scale bioreactors. Similar approaches may be used to produce other recombinant proteins and cytochromes in particular.

## 2. Results

When optimizing the production of recombinant proteins, the goal is to produce the highest amount of functional protein per time and per volume, which implies that a high cell density and high protein concentration should be reached. The impact of several upstream conditions on cyt b5 production was assessed at micro- and lab-scale reactors.

### 2.1. Media Optimization Using Microtitre Plates

A generic medium containing peptone, yeast extract, glucose, and sodium chloride, supplemented with ampicillin, was reported previously for the production of cytochrome b5 using *E. coli* TB-1 [26,27].

**Figure 1:**
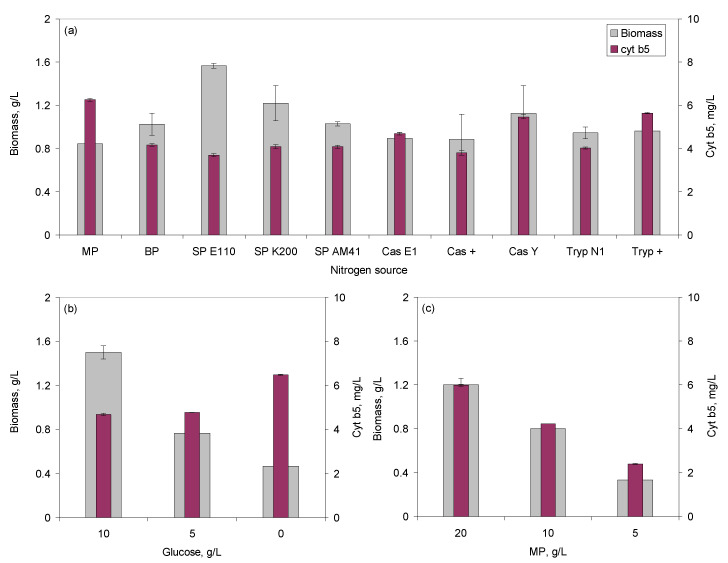
Biomass and cytochrome b5 production according to the nitrogen source (**a**), and to the initial glucose (**b**) and meat peptone (**c**) concentrations used.

In the present study, the effect of 10 peptones on the amount of both biomass and cyt b5 produced was evaluated in microtitre plates (MTP) with oxygen online monitoring. The highest biomass concentration, 0.78 g/L, was observed when soy peptone (SP) E110 was used as nitrogen source, whilst the highest cyt b5 concentration, 3.1 mg/L, was observed with meat peptone (MP; Figure 1a). The latter allowed the highest product to biomass yield, Y_P/X_, reaching 7.4 mg/g when the cells grew in the 24-well MTP. As seen previously for lactic acid bacteria [38], none of the peptones was able to maximize simultaneously cell growth and production.

When 10 g/L of MP and 5 g/L of yeast extract were used in the growth medium, and variable amounts of glucose were added, biomass concentration increased with glucose concentration whilst the reverse was observed with cyt b5 (Figure 1b). Without glucose, the Y_P/X_ reached 13.9 mg/g. The effect of the initial peptone concentration was also evaluated in media without yeast extract and glucose, but containing 5 g/L ammonium sulfate. In this case, Y_P/X_ varied between 4.9 and 7.2 mg/g when the initial MP concentration varied between 20 and 5 g/L (Figure 1c).

For the following experiments on a larger scale, MP was chosen as nitrogen source.

### 2.2. Scale-Up to Bioreactor

**Figure 2:**
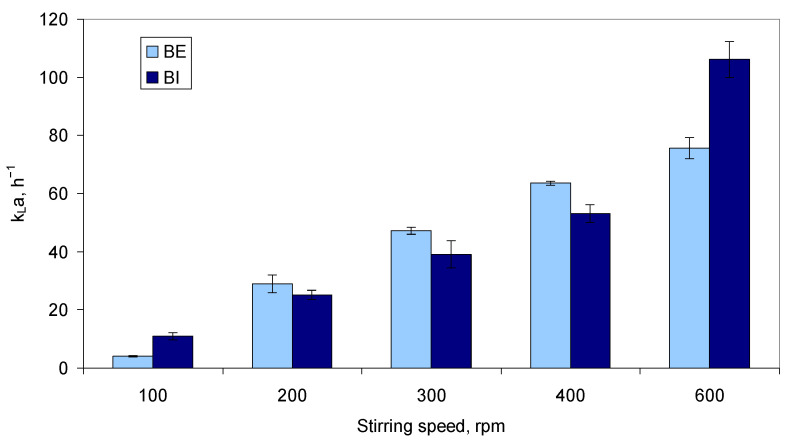
Volumetric mass transfer coefficient, *k_L_a*, attained at different stirring speeds in the bioreactors used. Values determined in the absence of cells with an air flow rate of 1 vvm.

The production of cyt b5 was scaled-up to two 2.6 L and two 2.5 L bioreactors, with different geometries (Table 1). This influenced the mass transfer coefficient, *k_L_a*. In the present study, *k_L_a* was determined using a dynamic method for several stirring speeds, as detailed in Materials and Methods. The value of *k_L_a* varied with the geometry of the bioreactor and increased with increasing stirring speeds (Figure 2). BE allowed *k_L_a* values 15–20% higher than BI for stirring speeds of 200–400 rpm. BI allowed a 2.7-fold higher *k_L_a* at 100 rpm and a 1.4-fold higher *k_L_a* at 600 rpm than BE bioreactors.

#### 2.2.1. Effect of Stirring, Air Flow, and PID Control

**Figure 3:**
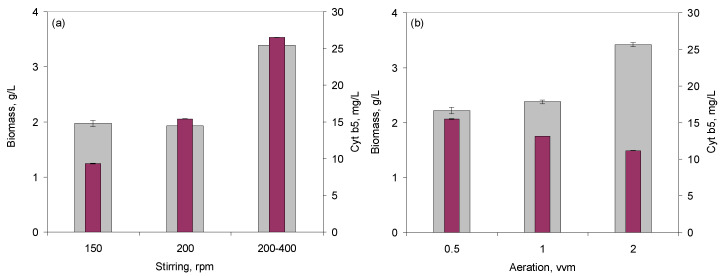
(**a**) Influence of stirring speed on biomass and cytochrome b5 production in the BE bioreactor. The aeration was set at 0.5 vvm, and dissolved oxygen was set at 2%, controlled by “cascade” mode, with stirring kept between 200–400 rpm. (**b**) Influence of air flow rate on biomass and cytochrome b5 production in the BI bioreactor. The dissolved oxygen was set at 2%, controlled by “cascade” mode, with stirring kept between 200–400 rpm.

The effect of dissolved oxygen on cell density and cyt b5 productivity was assessed by changes in the stirring and air flow rates and in the speed of response of the control system. Both biomass and cyt b5 concentrations increased when the concentration of dissolved oxygen in the growth medium was controlled by “cascade”, with stirring speeds raging between 200 and 400 rpm, when compared to fixed stirring speeds (Figure 3a). “Cascade” control increases the impeller speed to break air bubbles and force more oxygen into solution as cellular growth demand more oxygen. When the air flow rate into the fermenter was increased from 0.5 to 2 vvm, biomass concentration increased 54.0%, but cyt b5 concentration decreased 27.9% (Figure 3b).

**Figure 4:**
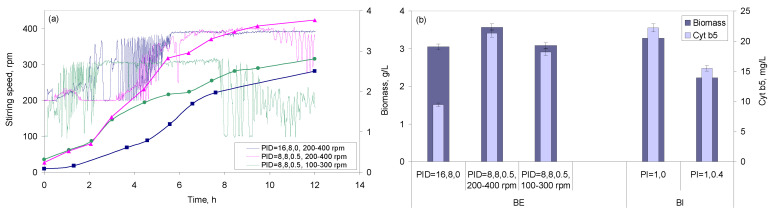
Effect of PID controller parameters on biomass and cyt b5 production. Initial glucose concentration 10 g/L, aeration set at 0.5 vvm, and dissolved oxygen at 2%. (**a**) Variation of stirring speed with PID parameters in the BE and corresponding biomass profile observed along with the fermentation. In blue: PID set at 16, 8, 0; cascade mode with stirring speed set between 200 and 400 rpm; biomass data in squares. In pink: PID set at 8, 8, 0.5; cascade mode with stirring speed set between 200 and 400 rpm; biomass data in triangles. In green: PID set at 8, 8, 0.5; cascade mode with stirring speed set between 100 and 300 rpm; biomass data in circles. (**b**) Influence of PID parameters and stirring speed on biomass and cyt b5 concentrations attained in the BE and BI. In the BI, the cascade mode was set with stirring speed that varied between 200 and 400 rpm.

Usually, during fermentations, oxygen must be supplied continuously at a rate similar to the consumption rate. In the present study, a balance between the oxygen necessary for cell growth and for cytochrome production was established, since opposite effects were observed (Figure 3). When the dissolved oxygen concentration in the growth medium depends on stirring speed, the response of the oxygen sensor may be fined-tuned by a proportional-integral-differential (PID) controller. In the present study, the values for the PID parameters were changed as follows: P between 16–8 for BE and 5–1 for BI; I between 16 and 4 for BE and 0.5 and 0 for BI; D between 0.5 and 0 for BE (not available for BI). The highest biomass concentrations in the BE were attained when PID was set at “8, 8, 0.5” and stirring speed allowed to vary between 200 and 400 rpm for a given air flow (Figure 4). These parameters also allowed the highest concentration of cyt b5 in the BE (Figure 4b), and a Y_P/X_ of 6.0. The same PID parameters, but with stirring speed varying between 100 and 300 rpm, resulted in a 13.7% lower concentration of biomass, a 14.7% lower concentration of cyt b5, but only a 1.1% decrease in Y_P/X_ (Figure 4b). In the case of BI, the highest concentrations of both biomass and cyt b5 were observed for PI set at “1, 0” (Figure 4b). An increase of the integral parameter to 0.4 resulted in a 32.2 and 30.3% decrease in biomass and cyt b5 concentrations, respectively, but in a 2.9% increase in Y_P/X_.

#### 2.2.2. Effect of Glucose Concentration

**Figure 5:**
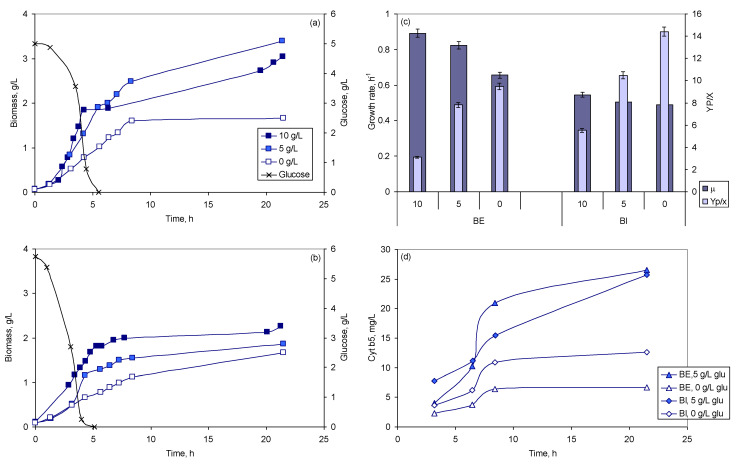
Effect of initial glucose concentration on biomass and cyt b5 production in BE (**a**,**d**) and BI (**b**,**d**), and respective growth rate and product to biomass yield (**c**). The maximum standard deviation observed was 10% of the mean value represented.

As observed in MTP, the concentration of *E. coli* TB-1 biomass attained, and the growth rate, increased with increasing concentrations of glucose in both types of bioreactors used (Figure 1b and Figure 5a–c). When glucose was depleted in the growth media, a maximum of 1.7 g/L of biomass was attained in the BI, and a biomass concentration of 1.8 g/L was reached in the BE, for an initial glucose concentration of 10 g/L. Diauxic growth was observed in the cultures grown with 5 and 10 g/L of glucose. MP and yeast extract should have been used as carbon source, besides being nitrogen sources, under these conditions and also in the cultures grown in the absence of glucose (Figure 5a,b).

Higher concentrations of cyt b5 were attained at 5 g/L of glucose when compared with cultures without glucose in both bioreactors (Figure 5d). However, a 3.0- and a 2.6-fold increase in Y_P/X_ were observed, respectively, for BE and BI when glucose was absent compared to an initial concentration of 10 g/L (Figure 5b).

#### 2.2.3. Effect of pH

**Figure 6:**
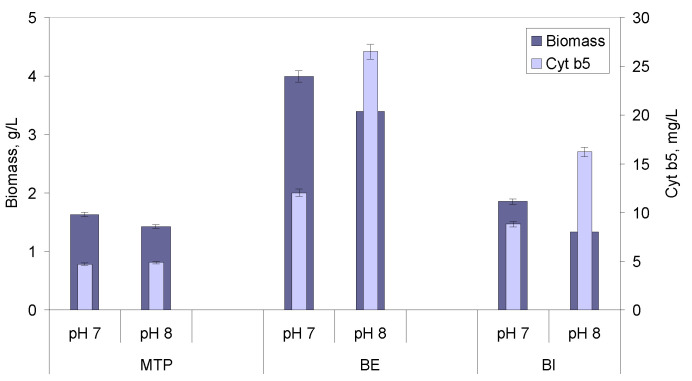
Effect of pH of the growth media on biomass and cyt b5 production by *E. coli* TB-1 cells. The initial glucose concentration of 5 g/L. The dissolved oxygen was set at 2% in “cascade” mode with stirring kept between 200–400 rpm.

The biomass concentration decreased when the growth was carried out at pH 8, when compared to growth performed at pH 7, when the cells grew in MTP and in the BE and BI bioreactors (Figure 6). However, the converse was observed for the concentration of product: cyt b5 concentration increased with pH values. A maximum concentration of biomass of 4.0 g/L was attained at pH 7 in the BE bioreactor, but a maximum cyt b5 concentration of 26.5 g/L was achieved at pH 8 in the same bioreactor. A 2.6-fold increase in Yx/p was observed between results obtained at pH 8 and pH 7 in both bioreactors.

#### 2.2.4. Multivariate Analysis of the Different Conditions

In the previous sub-sections, it was clear that both *E. coli* TB-1 biomass and cyt b5 production was influenced by several parameters, which may have opposite effects. To assess the best combination of conditions, 28 fermentations were carried out—14 in the BE bioreactor and 14 in the BI. The parameters and conditions tested were the following: The model of bioreactor represented by the height/diameter ratio of BE and BI; air flow was set at 0.5, 1, or 2 vvm; agitation was controlled by dissolved oxygen concentration in “cascade” mode, or it was set at 150 or 200 rpm; pH was set at 7.0 or 8.0; the initial glucose concentrations tested were 0, 5, 7.5 and 10 g/L; ampicillin was tested at 0 and 100 µg/mL; and the PID parameters were set at “1, 0, 0”, “0.15, 0.13, 0”, or “1, 0.4, 0” in the assays performed in the BI, and at “16, 8, 0”, or “8, 8, 0.5” for the assays carried out in the BE bioreactor. Therefore, multivariate analysis is necessary to determine the best set of parameters to allow the highest product yields.

Partial Least Squares (PLS) is a statistical method that allows the construction of predictive models when the variables or factors in a system are many and highly collinear [39,40]. PLS reduces the number of predictive factors which are calculated to maximize the covariance between the original variables.

**Figure 7:**
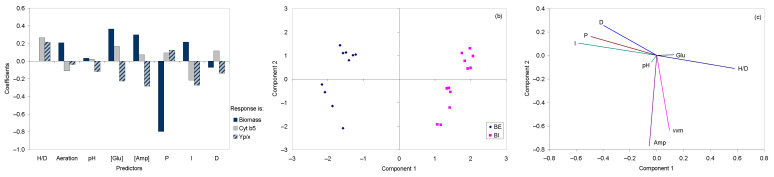
Partial least squares analysis. (**a**) Regression coefficients using as response biomass and cyt b5 concentrations, and Y_P/X_. Score (**b**) and loading (**c**) plots of PLS using Y_P/X_ as response.

In the present study, PLS was carried out using a matrix containing the parameters used in the 28 fermentations, and as response array, the respective concentration of biomass, cyt b5 or Y_X/P_ were used. When the concentration of biomass was used as response, the highest coefficients of the model was observed for H/D (−0.86) and P (−0.80), whilst the coefficients for glucose and ampicillin concentrations, I and aeration parameters were between 0.21 and 0.36 (Figure 7a). The values for D and pH presented coefficients of –0.07 and 0.03, respectively.

The coefficients of the PLS when cyt b5 concentration was set as the response was also highest for H/D (0.26), followed by the I parameter (−0.22). Glucose concentration presented a coefficient of 0.17, the D parameter of 0.11, and the aeration rate had a coefficient of −0.11. The ampicillin concentration and the parameter P presented coefficients of 0.07 and 0.09, respectively.

Since the coefficients of some parameters presented different values and opposite signals when the response of the PLS model was biomass or cyt b5 concentrations, the PLS was calculated using Y_P/S_ as response. In this case, ampicillin concentration and H/D presented the highest coefficients at −0.28 and 0.22, respectively. The concentrations of glucose, and the values for I, D, and pH presented negative coefficients between −0.12 and −0.27. The coefficient for the aeration rate was −0.03.

When comparing the three PLS models, it is curious to observe that the H/D ratio of the bioreactors was the most influential parameter (Figure 7a). In fact, when the scores of the model with Y_P/X_ as response are represented in the plane formed by components 1 and 2 of the model, which represent 58.3% of the variance, a clear clustering of the data according to the bioreactor used is observed (Figure 7b). The data produced in the BI bioreactors are presented on the right-hand side of the chart, where component 1 is positive, whilst the data produced in BE bioreactors are on the left-hand side.

When comparing the loading plot (Figure 7c) and the score plot (Figure 7b) of the model, it’s possible to infer the contribution of each variable in the model with Y_P/X_ as response. The relative contribution of each variable to the components (loadings) is directly proportional to the projection of the length of the variable vector on the principal components. The first component, which represents 39,4% of the total data variance, is mainly a function of H/D and PID (Figure 7c). The second component, which explains 18.9% of the total data variance, has major contributions from the concentration of ampicillin and aeration rate (Figure 7c). The fermentations carried out in the BI bioreactors were located mainly on the part of the plane where H/D was the parameter influencing most the PLS model (Figure 7b,c). On the other hand, fermentations carried out in the BE bioreactors were located where the values of PID control had increased importance.

To test the knowledge acquired with the PLS model, four fermentations were conducted both in the BE and BI bioreactors, under the conditions that should lead to the highest concentration of cyt b5 and Y_P/X_. Comparing to the average of the fermentations used for the PLS model for each bioreactor type, the cyt b5 concentration increased 1.7- and 2.5-fold for the BE and 2.4- and 4.9-fold for the BI, for conditions 1 and 2, respectively (Table 2). The highest concentration of cyt b5 attained in the BE was 30.70 mg/L, whilst in the BI bioreactor, 72.72 mg/L could be attained. Under conditions 2, a 2.6-fold increase in Y_P/X_ was observed in the BE bioreactor, and a 3.6-fold increase in the BI, when compared to average values attained in the previous cultures for each reactor type (Table 2).

## 3. Discussion

It is commonly known that cultivation parameters, such as media composition, pH, stirring and aeration conditions, temperature, cell density, substrate and feeding strategies, concentration of inducers, and time of induction, affect both cell density and protein expression levels [33,34,41]. Peptones, besides being used as nitrogen source, play an important role during the growth of bacterial strains, stabilizing enzyme activity [42], and influencing cell surface properties [43]. Growth kinetics and metabolism of the culture is, thus, dependent on the composition of the peptone used [38]. Commercial peptones for microbial culture media are mainly derived from casein, soy, and meat. Tryptone, for example, is produced by the digestion of casein with the pancreatic extract. It was shown previously that, under oxygen limiting conditions, the content of cytochrome b_558/566_ double or tripled when yeast extract was substituted by peptone or by selected amino acids [44]. In the present study, the type and concentration of peptones influenced both cell density and cyt b5 production (Figure 1). MP allowed the highest Y_P/X_. This selection could be made using a high throughput system, and it showed for the first time that conditions leading to high biomass concentrations could inhibit cyt b5 production and vice versa, e.g., increasing glucose concentrations allowed higher biomass, but lower cyt b5 production.

The production of cyt b5 was also found to be highly dependent on the dissolved oxygen concentration. Belo and Mota showed that increased cyt b5 productivity could be achieved by increasing the air pressure inside the bioreactor to 0.48 MPa when compared to increasing the stirring rate to 500 rpm, since *E. coli* TB-1 cells are sensitive to high shear stress caused by high agitation speeds [26]. In this case, productivity reached 2.4 mg_cyt b5_/L.h. The authors suggested that the oxygen transfer rates could be controlled by manipulation of the inlet air pressure, which resulted in increased cyt b5 productivity. As shown by other studies, the amount of available oxygen must be balanced between cell growth and protein yields, since higher oxygen concentration may favor cell growth, but increase oxidative stress within the cells, whilst anoxic conditions may limit amino acid production and plasmid stability [45,46,47]. In the present study, the geometry and *k_L_a* values reached in the two types of lab-scale bioreactors used, the aeration rate, the stirring speeds, and the PID parameters to control the dissolved oxygen concentration influenced both the cell density and the yield of cyt b5 reached (Figure 2, Figure 3 and Figure 4).

Since other parameters, such as the initial glucose concentration and pH, produced the opposite effect on biomass and cyt b5 concentrations, statistical multivariate analysis tools were applied for the interpretation of the most critical parameters. Statistical techniques have been successfully used for the understanding of biological systems and optimization of bioprocesses [48,49,50]. In particular, PLS was used to model the influence of the different parameters on biomass and cyt b5 concentrations, and on product yield. In this statistical technique, data from multiple batches are projected into a lower-dimensional space, and latent variables are calculated to maximize the covariance between the scores of an independent matrix, containing the original variables, and the scores of a dependent array of responses, making it suitable to model batch and fed-batch processes [51,52]. The three PLS models developed in the current study indicated that the H/D of the bioreactor and PID control of dissolved oxygen concentration had a significant impact on the production of biomass, cyt b5, and respective production yield. In fermenters, regulation of dissolved oxygen concentration may be fine-tuned by a PID algorithm, especially when oxygen supply is controlled by stirring speed [53,54]. Adjustment of the three parameters may improve the regulation for low oxygen concentrations [52]. It has been suggested that classic PID controllers cannot be used to accurately control dissolved oxygen concentration in high-performance processes, such as during the production of recombinant proteins, since these systems are rapidly changing [55,56]. Interactive or dynamic controllers could improve cell response. By using the information acquired with the PLS model, the best conditions to improve Y_P/X_ could be found (Table 2). In the BE bioreactor, a maximum of 30.70 mg/L of cyt b5 could be thus produced, corresponding to a yield of 11.08 mg/g. In the BI bioreactor, a maximum of biomass and cyt b5 concentrations of respectively 4.69 g/L and 72.72 mg/mL could be attained. A maximum yield of 24.97 mg/g was attained, corresponding to productivity of 3.4 mg/L.h. This value is 41% larger than the productivity reported by Belo and Mota [26]. Thus, the present work clearly shows that multivariate data, treated by statistical techniques, provide better information than factor-at-a-time analysis. This may be used for developing other systems to produce recombinant proteins.

## 4. Materials and Methods

### 4.1. Bacterial Strain and Growth Conditions

The *Escherichia coli* TB-1 strain was genetically transformed by introducing the PUC13 plasmid with ampicillin resistance gene to produce the rat hepatic cyt b5 heme protein [6]. The cells are maintained in cryotubes at −80 °C with 20% glycerol. To identify the transformants containing the cyt b5 sequences, the transformed TB-1 cells were grown on LB agar plates (amp^+^) at 37 °C for 24 h. The pink/red colonies, indicating cyt b5 accumulation, were transferred to 250 mL Erlenmeyers containing 150 mL of growth medium. The complex medium contained the following compounds according to Santos et al. [27]: 5 g/L of yeast extract, 5 g/L of sodium chloride, 10 g/L of glucose, 10 g/L of peptone, and 100 µg/mL of ampicillin.

### 4.2. Effect of Media Composition and pH

The effect of media composition on biomass and cyt b5 production was assessed in Deep Well OxoDish^®^ OD24-DW plates (PreSens Precision Sensing GmbH, Regensburg, Germany) containing 2 mL of growth medium (Table 3). The ampicillin concentration was 100 µg/mL or was absent in some bioreactor experiments to test its influence. Growth started by the addition of 10% (*v*/*v*) inoculum from an overnight grown culture, and was carried out at 37 °C and 150 rpm in an orbital incubator (Agitorb 200, from Aralab, Rio de Mouro, Portugal). Bacterial growth was assessed by real-time measurement of the dissolved oxygen concentration, as previously described [57]. Data acquisition was performed by the software SDR_v37 also from PreSens. According to the manufacturer, the Deep Well microtiter plates have a resolution of ±0.4% O_2_, a precision of ±1% O_2_, and a drift <0.2% O_2_ within one week.

The influence of the pH on biomass and product yields was also assessed for the medium selected in the previous experiment. Growth was promoted in HydroDish^®^HD6 plates. A control was carried out without pH correction, and two other experiments were performed with the initial pH corrected to 7 and 8. Data acquisition was performed by the software SDR_v37 also from PreSens. According to the manufacturer, the 6-well plates have a resolution of ±0.05 pH and precision of ±0.2 pH, both at pH 7, and a drift < 0.1 pH within one week.

Both Deep Well OxoDish^®^ OD24-DW and HydroDish^®^HD6 plates were closed with sealing tape, optically clear (Sarstedt, Nümbrecht, Germany), to prevent evaporation. All experiments were carried out at least in duplicate.

### 4.3. Cell Growth in 2 L Bioreactors

The effect of glucose concentration, pH, and dissolved oxygen control (by optimizing the proportional-integral-derivative, PID, controller system) on cell growth and product production was determined in 24 h batch cultures. Four geometrically different 2 L bioreactors were used: Two Fermac 360 bioreactors (Electrolab Biotech, Gloucestershire, UK), with 1.5 L of working volume; and two Minifors bioreactors (Infors HT, Bottmingen, Switzerland), with a working volume of 1.2 L. Batch cultures were performed at 37 °C, and at pH 7 and 8. The pH was controlled automatically by the addition of 2N NaOH (Panreac, Germany) or 2N of H_2_SO_4_ (Sigma-Aldrich, USA). To keep the dissolved oxygen (DO) at a set-point of 2% saturation, a DO cascade controlled by each bioreactor was used, where stirring was kept between 100–300 or 200–400 rpm. Different air flows, between 0.5 and 2 vvm, were tested.

The oxygen mass transfer coefficients were determined by the dynamic method at each stirring speed, using a Mettler polarographic probe (Mettler-Toledo, LLC, Columbus, OH, USA) to measure DO, and nitrogen to deoxygenate the liquid by stripping [58]. To optimize the PID parameters, a “bump test” was performed during mid-exponential growth phase. For that, the cascade mode operating was turned off, and the agitation speed was changed as a step-response to assess the “system” behavior in terms of oxygen variation.

### 4.4. Analytical Methods

Oxygen mass transfer coefficients were determined in the bioreactors without biological consumption of oxygen, according to Garcia-Ochoa and Gomez [59]. Briefly, deaeration of the media was accomplished by flushing air with nitrogen. After complete deaeration, the air was introduced into the medium, and the concentration of oxygen along time was registered, as well as the saturation concentration. *k_L_a* was determined by Equation (1)

(1) $$\frac{dC}{dt}=k_{L}a\;(\overset{-}{C}-C)$$

where $\overset{-}{C}$ is the oxygen saturation concentration in the bulk medium at equilibrium with the bulk gas phase, and *C* is the oxygen concentration at time *t*.

### 4.5. Analytical Methods

Growth of *E. coli* TB-1 cells was monitored off-line by optical density measurements at 600 nm in a double beam spectrophotometer (T80 from PG Instruments, Leicestershire, UK). Glucose concentration was determined by HPLC analysis using a Hitachi LaChrom Elite system (Hitachi Ltd., Tokyo, Japan), equipped with a Rezex ROA-Organic acid H^+^ 8% (300 mm × 7.8 mm) column (Phenomenex, Torrance, CA, USA), and a Hitachi L-2490 refractive index detector. The column was operated at 65 °C, with a flow rate of 0.5 mL/min of 5 mM H_2_SO_4_ solution, and the sample volume was 20 µL [60].

The cyt b5 concentration was determined after cell disruption. For this, 4 mL samples were taken from the culture medium, and *E. coli* cells were harvested by centrifugation at 11,500× *g* for 5 min, and washed twice with 20 mM phosphate buffer, pH 7.0. The cell pellet was stored at −20 °C, since freeze/thawing prior to sonication improved cyt b5 recovery [27]. *E. coli* TB-1 cells were resuspended in 20 mM phosphate buffer pH 7.0 and disrupted by sonication (on a Sonopuls HD 3200 from Bandelin electronic GmbH and Co. KG, Berlin, Germany) using an MS 72 titanium probe, for 3 min at 30 W with on/off cycles of 10 s, in a cylindrical glass vial place over an ice bath. After disruption, the cells suspensions were centrifuged at 19,000× *g* for 30 min. Cyt b5 concentration was determined by optical density measurements at 410 nm in the previously mentioned double beam spectrophotometer, using the Lambert–Beer law with an extinction coefficient of 130 nM^−1^ cm^−1^ [6].

### 4.6. Multivariate Analysis

Partial Least Squares (PLS) models were calculated using the statistical software Minitab^®^ Release 14.1 from Minitab, LLC (State College, Pennsylvania, PA, USA). As PLS analysis assumes variance homogeneity, the data was scaled by subtracting the mean and dividing by the standard deviation.

## Figures and Tables

**Table 1:** Geometric parameters of the bioreactors used in this study.

Bioreactor Code	BE	BI
Model	Fermac 360 (Electrolab)	Minifors (Infors HT)
Volume (L)	2.6	2.5
Diameter (cm)	12.0	11.0
Height (cm)	23.0	26.3
Height/Diameter ratio	1.9	2.4
*Baffles*		
Number	2	3
Height (cm)	15.4	21.0
Width (cm)	1.0	1.5
*Impellers*		
Type	Rushton turbine	Rushton turbine
Number	2	2
Diameter (cm)	5.7	4.7
Blade height (cm)	1.6	1.1
Blade width (cm)	1.7	1.0

**Table 2:** Selected conditions used to produce cyt b5 by *E. coli* TB-1 cells in the bioreactors BE and BI.

Bioreactor/Condition	H/D	vvm	Stirring	pH	Glu, g/L	Amp, ug/mL	P	I	D	X, g/L	Cit, mg/L	Y_P/X_
BE/1	1.9	0.5	cascade	7	10	100	8	8	0.5	3.57	21.28	5.96
BE/2	1.9	1	cascade	7	10	100	16	8	0	2.77	30.70	11.08
BI/1	2.4	0.5	cascade	7	10	100	1	0	0	4.69	36.02	7.68
BI/2	2.4	1	cascade	7	10	100	1	0	0	2.91	72.72	24.97

**Table 3:** Identification of the compounds, including the different peptones, used in the growth medium of *E. coli* TB-1.

Name	Abbreviation	Concentration (g/L)	Supplier
Bacto^TM^ Yeast Extract	Yeast extract	5	BD Biosciences, San Jose, CA, USA
Sodium chloride	NaCl	5	Panreac, AppliChem GmbH, Darmstadt, Germany
D(+)-Glucose	Glu	10	Merck, Darmstadt, Germany
Ampicillin sodium salt	Amp	0.1	Sigma-Aldrich, St. Louis, MO, USA
*Nitrogen source tested*			
Meat Peptone, enzymatic digest for microbiology	MP	10	Millipore Sigma, St. Louis, MO, USA
Casein Yeast Peptone	Cas Y		
Bacteriological Peptone	BP		Thermo Fisher Scientific, Inc., Waltham, MA, USA
Soy Peptone	SP E110		OrganoTechnie, SAS, La Courneuve, France
	SP K200		
	SP AM41		
Casein Peptone	Cas E1		
	Cas +		
Tryptone	Tryp N1		
	Tryp +		

## Data Availability

The data presented in this study are available on request from the corresponding author.

## Sample Availability

Samples of the compounds are not available from the authors.
